# Adaptations of seal louse nits to underwater life: morphology, respiration and attachment

**DOI:** 10.1007/s00114-026-02095-2

**Published:** 2026-04-10

**Authors:** Anika Preuss, Thomas van de Kamp, Stanislav N. Gorb

**Affiliations:** 1 Department of Functional Morphology and Biomechanics, Zoological Institute, Kiel University, Kiel, Germany; 2 Karlsruhe Institute of Technology (KIT), Institute for Photon Science and Synchrotron Radiation (IPS), Hermann-von-Helmholtz-Platz 1, Eggenstein-Leopoldshafen, 76344 Germany; 3 Laboratory for Applications of Synchrotron Radiation (LAS), Karlsruhe Institute of Technology (KIT), Kaiserstr.12, Karlsruhe, 76131 Germany

**Keywords:** Insect, Egg, Adhesion, Glue, Biomechanics, Anoplura, Insecta, *Echinophthirius horridus*

## Abstract

Marine mammals host a variety of parasites, engaged in continuous evolutionary competition. Our understanding of parasitic insects associated with these mammals, specifically their physiological adaptations to the marine environment, remains limited. Among thirteen insect species capable of enduring extended underwater periods in open oceans is the seal louse, *Echinophthirius horridus*, which infests true seals. This species relies on adaptations to survive extreme conditions such as hypoxia, temperature variations, hydrostatic pressure, and drag forces during host dives. These conditions affect both adult lice and louse embryos within their nits. Questions arise about how these early stages survive underwater, respire inside the eggs during dives, and what limits their survival. Using CLSM, SEM, and synchrotron X-ray microtomography, we identified morphological structures consistent with a potentially specialized respiratory system in the nits, a multi-layered eggshell and structural features that may help limit water entry. Adhesive force tests showed that the nit sheath attaching eggs to seal hair is not the primary vulnerability during host dives; rather, prolonged submersion likely imposes oxygen limitation over time. This research enhances our understanding of ectoparasite-host dynamics in marine mammals and demonstrates remarkable adaptability of insects to challenging aquatic niches.

**Supplementary Information:**

The online version contains supplementary material available at 10.1007/s00114-026-02095-2.

## Introduction

Living underwater is a challenging task: hypoxia, currents, high pressure, fluctuating temperatures, and food shortages make it difficult for many creatures to survive in this challenging environment. It is therefore not surprising that also in the insect world only 75,000 of the 1 million known insect species are found in the aquatic environment (Grimaldi and Engel 2005; Balian et al. 2008; Zhang 2011; Stork 2018). In the marine environment, there are even fewer: only 1400 insect species are capable of surviving here mainly inhabiting coastal areas (Ruxton and Humphries 2008; Cheng 2009). In the open sea, only 13 insect species have been found capable of withstanding the harsh conditions in this habitat. And all these 13 species belong to the sucking lice of the Echinophthiriidae family (Anoplura; Insecta), which are parasites (Durden and Musser 1994; Leonardi and Palma 2013). One example is the ectoparasitic seal louse, *Echinophthirius horridus*, which lives in the fur of harbor seals (*Phoca vitulina*) and grey seals (*Halichoerus grypus*) and feeds on the blood of its host animals (Grzimek 1990; Durden and Musser 1994; Leonardi and Palma 2013). It shows extreme adaptations to its challenging living environment. For example, it can withstand pressures of up to 5883.96 kPa (Leonardi et al. 2020) and has a very sophisticated spiracle closing mechanism, to prevent the ingress of water during the seal’s dives (Preuss et al. 2025a), which can be up to 600 m deep and last 20–35 min (Hastings et al. 2004; Eguchi and Harvey 2006; Rosing-Asvid et al. 2020), while it uses skin and pigment respiration and the air reservoir in its tracheal system to avoid drowning underwater (Leonardi et al. 2025; Preuss et al. 2025a). Additionally, it has the highest attachment force ever measured in insects, due to a specialized carabiner hook system that allows it to cling to the fur (Preuss et al. 2024; Preuss et al. 2025b).

The entire life cycle takes place on the host animal, and the louse is highly adapted to its host’s lifestyle: echinophthiriids are capable of reproduction, hatching, and dispersal exclusively during their hosts’ terrestrial periods (haul-outs), requiring a precise synchronization of their life cycle with that of their hosts (Murray et al. 1965; Kim 1971, 1972). However, harbor seals spend on average only about 17% of their time on land (Hastings et al. 2004; Eguchi and Harvey 2006; Blundell and Pendleton 2015) and may even travel offshore for several days (Thompson et al. 1998; Adelung et al. 2006; Wilson et al. 2015; Vance et al. 2021), as they spend most of their time in the water foraging to obtain the approximately 4.6 kg of fish they require per day (Aarts et al. 2019). Consequently, lice reproduction must be closely synchronized with the seals’ breeding period, during which haul-out durations are substantially longer (Huber et al. 2006; London et al. 2012), which in turn likely limits the number of generations these lice can produce (Aznar et al. 2009). The reason for the necessity of this synchronization is, on the one hand, due to the fact that in several echinophthiriid species, lice have been reported to reduce their metabolism during host dives to such an extent that they fall into a state of akinesis (Kim 1975; Koštál 2006; Leonardi and Lazzari 2014; Leonardi et al. 2020) and, accordingly, mating and egg laying under water is impossible (Murray et al. 1965; Leonardi and Lazzari 2014). However, in a recently published study on *E. horridus*, it was found that this louse reproduces throughout the year regardless of the reproductive behavior of its hosts, as nits were found on the animals at all times of the year. Yet, it is not known whether these were still viable (Herzog et al. 2024a, b), although other studies on echinophthiriids parasitizing other marine mammals suggest that the eggs would not survive under water in the long term (Murray et al. 1965; Leonardi and Lazzari 2014). If the latter is true, the obvious question is why. Is it due to the lack of oxygen supply or does the nit sheath, with which the nits of lice are fixed to the host hair by the adults, come off under water?

In general, first instar nymphs (N1) of other seal lice species can remain submerged for shorter durations without replenishing oxygen and maintain viability, possibly due to the fact that the respiratory system of smaller insects or those in earlier developmental stages is not as fully developed as in mature specimens (Leonardi and Lazzari 2014; Polilov 2015, 2016). At a very early stage of the louse development, it is therefore possible that their respiratory system is not yet up to the challenges of the marine underwater environment and they drown in the eggs. On the other hand, the smaller the animal actually is, the less specialized respiratory systems are usually required due to the scaling effects: the animals can potentially use cutaneous respiration (Polilov 2015; Propistsova et al. 2023). Therefore, another possibility might be that the adhesive binding of nits to seal hairs may lack long-term water resistance, potentially causing detachment over time. In land-dwelling lice, this adhesive is produced by the accessory gland and rapidly solidifies to secure eggs to hair or fibers (Park et al. 2019; Kim et al. 2021). Upon hardening, this substance forms a solid sheath encompassing both the hair and egg, commonly referred to as the nit sheath (Carter 1990). This mechanism provides attachment and protects the egg from external factors through a presumably waterproof substance (Burkhart et al. 1998). The embryo respires via an adhesive-free dome called an operculum including respiratory pores (aeropyles), shielded by the nit. Following an 8–10 day incubation, the egg hatches, and the louse nymph emerges, leaving its egg shell (Burkhart et al. 1998). While previously thought to be chitin-based, recent analyses of nits using histochemical techniques and flash pyrolysis gas chromatography/mass spectrometry indicate that they are composed of proteins, including transglutaminase (TG), which cross-links the nit sheath proteins, and additionally show a high contribution of saturated fatty acids such as C14 (myristic) and C16 (palmitic), along with other aliphatic substances like alkanols (Burkhart 1999; Park et al. 2019; Kim et al. 2021). These louse nit sheath proteins (LNSP) also function as nit adhesive (Park et al. 2019; Kim et al. 2021).

To investigate the factors limiting seal lice reproduction, this research aims to elucidate (i) the developmental process of *E. horridus* within the nit over time, (ii) the structural elements supporting its respiratory capacity inside the nit, and (iii) the impact of water on nit attachment to seal hair. The study employs advanced imaging techniques, including 3D reconstructions based on synchrotron X-ray microtomography (Prebus et al. 2023; Thakur et al. 2024; Bajerlein et al. 2025), confocal laser scanning microscopy (CLSM) (Michels and Gorb 2012; Appel et al. 2015), and cryo-scanning electron microscopy (Cryo-SEM) (Gorb et al. 2012; Gorb and Gorb 2022). Furthermore, we directly measured adhesive forces generated by the seal lice nits, when attached to the seal fur in dry condition or underwater. This approach offers new insights into the extreme adaptations, marine parasites underwent, when the ancestors of their recent hosts made transition from land to the sea during the Miocene, confronting these parasites with numerous novel challenges (Anderson 1982; Raga et al. 2009; Rybczynski et al. 2009).

## Materials and methods

### Animals

**Fig. 1:**
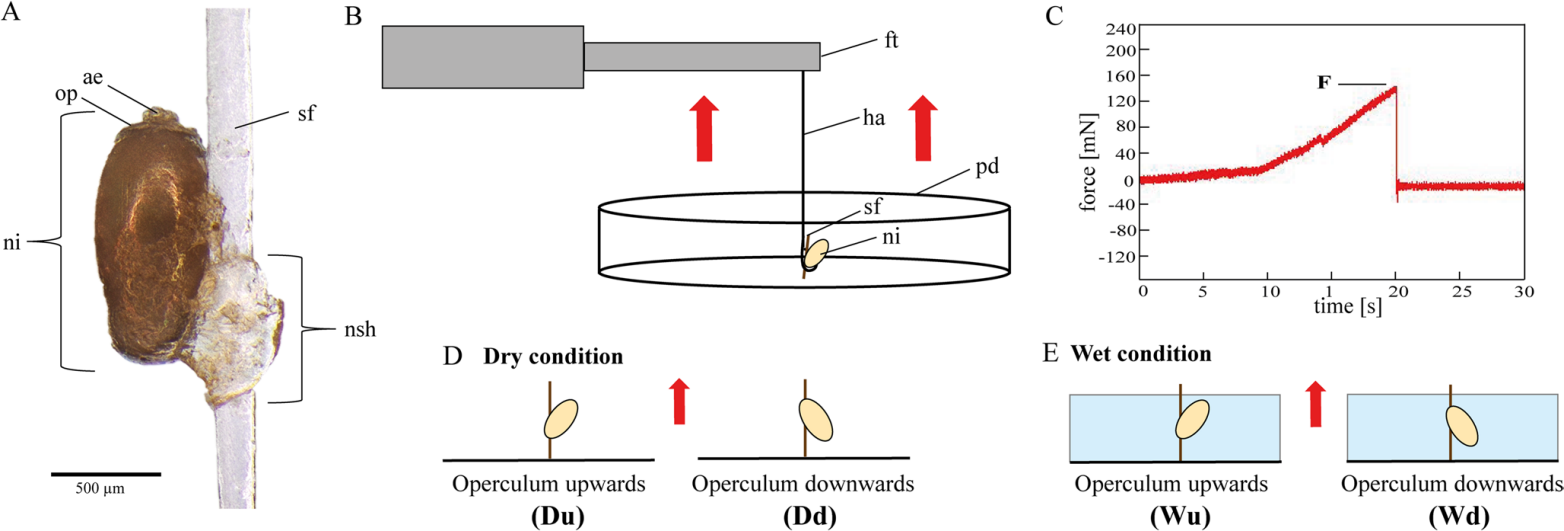
Experimental setup for adhesion measurements of seal louse nits on seal hair under different conditions. (**A**) Seal louse nit attached to seal fur. The seal louse nit (ni) is glued to the seal fur (sf) with the nit glue, forming the nit sheath (nsh) and has a visible operculum (op) including aeropyles (ae). (**B**) Experimental setup. The seal louse nit (ni) was fixed with a human hair (ha) to a force transducer (ft) and actively pulled off from seal fur (sf), which was fixed with glue to a Petri dish (pd). (**C**) Representative force-time curve showing the pull-off force at detachment (F). (**D**) Orientation of nits in dry condition during experiments. (**E**) Orientation of nits in wet condition during experiments

Seal louse nits (*E. horridus*; Anoplura; Insecta) (Fig. 1A) were obtained during necropsies of harbor seals (*P. vitulina*) and grey seals (*H. grypus*), which were discovered deceased or moribund along the Baltic Sea coast in Schleswig-Holstein between April and November 2022. The specimens examined in this investigation were derived from seals analyzed as part of monitoring programs within the stranding network of Schleswig-Holstein to assess their health status (Siebert et al. 2007; Herzog et al. 2024a, b). Skin samples, including nits, were freshly frozen and stored at -20 °C. Seal louse nits were stored in 70% ethanol for CT-analysis. Ethical review and approval are not required for this study because all host animals were found dead, died naturally or were euthanized based on welfare grounds and none of the host animals was killed for the purpose of this study. The authors were not involved in host euthanasia as this was done by a third party (certified seal rangers) for external reasons unrelated to this study. We have complied with all relevant ethical regulations for animal use.

### Scanning electron microscopy (SEM)

Frozen seal louse nits (*n* = 5) were subjected to examination utilizing cryo-scanning electron microscopy (SEM). The methodology involved freezing the nits at -140 °C in a cryo stage preparation chamber (Gatan ALTO 2500 cryo preparation system, Gatan Inc., Abingdon, UK). Subsequently, the frozen samples were coated with a 6 nm layer of gold-palladium via sputter-coating. Observation of the samples was conducted in a frozen state using a cryo-SEM Hitachi S-4800 (Hitachi High-Technologies Corporation, Tokyo, Japan) at 3 kV accelerating voltage and − 120 °C. The resultant images were enhanced utilizing Adobe Photoshop CS6 (Adobe Photoshop CS, San José, USA) and Affinity Photo (Serif Ltd, Nottingham, UK).

### Confocal laser scanning microscopy (CLSM)

To conduct CLSM analysis, seal louse nits (*n* = 3) were immersed in glycerine (≥ 99.5%) and covered with a precision cover slip (thickness = 0.170 mm ± 0.005 mm, refractive index = 1.52550 ± 0.00015, Carl Zeiss Microscopy GmbH, Jena, Germany) prior to scanning. The samples’ autofluorescence was examined utilizing a Zeiss LSM 700 CLSM mounted on an upright Zeiss Axio Imager microscope (Carl Zeiss Microscopy GmbH, Jena, Germany). The analysis employed four solid-state lasers (wavelengths 405 nm, 488 nm, 555 nm, 639 nm) and their corresponding emission filters (BP420–480, LP490, LP560, LP640 nm). Following the methodology described by Michels and Gorb (2012), the 405 nm excitation and 420–480 nm emission filter usually highlights less sclerotized cuticle, potentially rich in resilin. Regions of higher sclerotization are identified using 488 nm and 555 nm laser excitations with filters that transmitted emission light above 490 nm and 560 nm respectively. The 639 nm laser excitation with a 640 nm long-path emission filter captures extended autofluorescence. Image projections were processed using ZEN 2008 software (www.zeiss.de/mikroskopie) and Adobe Photoshop CS6 (Adobe Photoshop CS, San José, USA) for qualitative, but not quantitative, analysis of cuticle composition (Andersen 1979; Vincent 2002; Michels and Gorb 2012; Büsse and Gorb 2018; Josten et al. 2022). 

### Synchrotron X-ray microtomography and 3D reconstruction

Samples of *E. horridus* (*n* = 2) were scanned at the IMAGE beamline (Cecilia et al. 2025) at KIT Light Source’s IMAGING Cluster in 70% ethanol. A polychromatic X-ray beam, produced by a superconducting wiggler (corresponding to a magnetic field B = 1 Tesla) was filtered with 10 mm pyrolytic graphite sheets, resulting in a spectrum with a peak at approximately 16.5 keV and having a full width at half maximum of approximately 11 keV. The setup included a rapid indirect detector system with a scintillator, visible light optics, a white beam microscope (Optique Peter, Lentilly, France) (Douissard et al. 2012), and a 12-bit pco.dimax high-speed camera (Excelitas PCO GmbH, Kelheim, Germany) featuring 2016 × 2016 pixels, each 11 μm in size. A 10x magnification resulted in an effective pixel size of 1.22 μm. Each scan consisted of 100 dark field images, 200 flat field images, and 3000 equiangularly distributed radiographic projections over 180° at 60 fps. The concert control system facilitated automated data acquisition (Vogelgesang et al. 2016). The UFO framework managed data processing, including dark and flat field correction and phase retrieval (Vogelgesang et al. 2012). The final 3D tomographic reconstruction was performed by tofu and included ring removal, 8-bit conversion and blending of the phase and absorption 3D reconstructions (Faragó et al. 2022).

Amira 6.2.0 (Thermo Fisher Scientific, Waltham, US) was utilized for data segmentation, while Blender 3.4 (Blender Foundation, Amsterdam, Netherlands) was employed for visualization and rendering purposes.

### Adhesive force measurement

To assess the maximum pull-off force (dependent on the failure mode; hereafter referred to as adhesive force for consistency) required to detach seal louse nits from seal fur, force measurements were conducted (Fig. 1B, C) using a BIOPAC MP 100 data acquisition system (BIOPAC System Inc, Goleta, USA) with a Fort100 force transducer (100 g capacity, World Precision Instruments Inc., Sarasota, USA). The force transducer was attached to a compact linear stage with a stepper motor (Physik Instrumente GmbH & Co. KG, Karlsruhe, Germany) for precise computer-controlled movement. A single seal hair with an attached nit was secured at the tip using a 2-component epoxy resin adhesive (R&G Faserverbundstoffe GmbH, Waldenbuch, Germany; tensile shear strength according to DIN 53281, aluminum (AlCuMg1): 9.3 MPa) in a Petri dish, which was subsequently filled with Baltic Sea water (18 psu). A human hair was connected to the force transducer and tied with a loop to the base of the nit, enabling vertical pulling of the nit from the seal fur (Fig. 1B).

The force transducer was moved vertically to apply tension to the human hair until the nit detached from the seal fur. All tested nits were attached to seal hairs in their natural orientation, with the operculum facing away from the host skin. To test potential effects of orientation, the hair was positioned either with the operculum facing upwards or downwards relative to the direction of the applied force. Four distinct scenarios were examined (Fig. 1D, E): (A) dry nits with upward-facing operculum (Du); (B) dry nits with downward-facing operculum (Dd); (C) wet nits with upward-facing operculum (Wu); and (D) wet nits with downward-facing operculum (Wd). For wet conditions, nits on the hair were submerged in Baltic Sea water for 2 h prior to underwater measurements.

Force-time curves were recorded using AcqKnowledge 3.7.0 software (BIOPAC System Inc, Goleta, USA) to determine maximum adhesive forces (Fig. 1C). To account for variations in contact area with the seal hair (*A*_*0*_) among nits, the adhesive strength ($\:{\sigma\:}_{c}$) was calculated based on the relationship between maximum adhesive force (*F*_*ad*_) and the contact area with the seal hair (*A*_*0*_). The contact area of the nit sheath with the seal hair was measured and calculated using a Keyence VR 3100 (Keyence Corporation, Osaka, Japan).

$$\:{\sigma\:}_{c}=\:\frac{{F}_{ad}}{{A}_{0}}$$

A total of 40 nits (10 per scenario) were tested, with adhesive forces recorded (original data is provided in Supplementary Data 1). Because the developmental stage of the nits cannot be reliably determined from external morphology alone, we assessed their appearance using light microscopy (Keyence VR 3100, Keyence Corporation, Osaka, Japan) during the size measurements. At this magnification, the developing embryo was often visible as a slightly brownish structure through the eggshell, which allowed us to select nits with a comparable developmental appearance for the experiments. As the adhesive force measurements primarily involve the interaction between the seal hair and the hardened nit sheath, which forms during oviposition and remains structurally stable thereafter, variation in embryo development is unlikely to have influenced the measured adhesive forces.

### Data analysis and statistics

We compared the adhesive force and adhesive strength among the four experimental scenarios (dry condition: nits with the operculum oriented upwards or downwards; wet condition: nits with the operculum oriented upwards or downwards) using a Kruskal–Wallis test with a significance level of α = 0.05. Normality of the data was assessed using the Shapiro–Wilk test. Given the relatively small sample size (*n* = 10 per group), non-parametric statistics were applied. Pairwise comparisons were performed using Dunn’s post hoc test with Bonferroni correction. All statistical analyses were conducted in RStudio (R version 4.2.1; R Core Team 2022). The R scripts are provided in the Supplementary Materials (S2).

## Results

### Outer morphology of seal louse nits

**Fig. 2:**
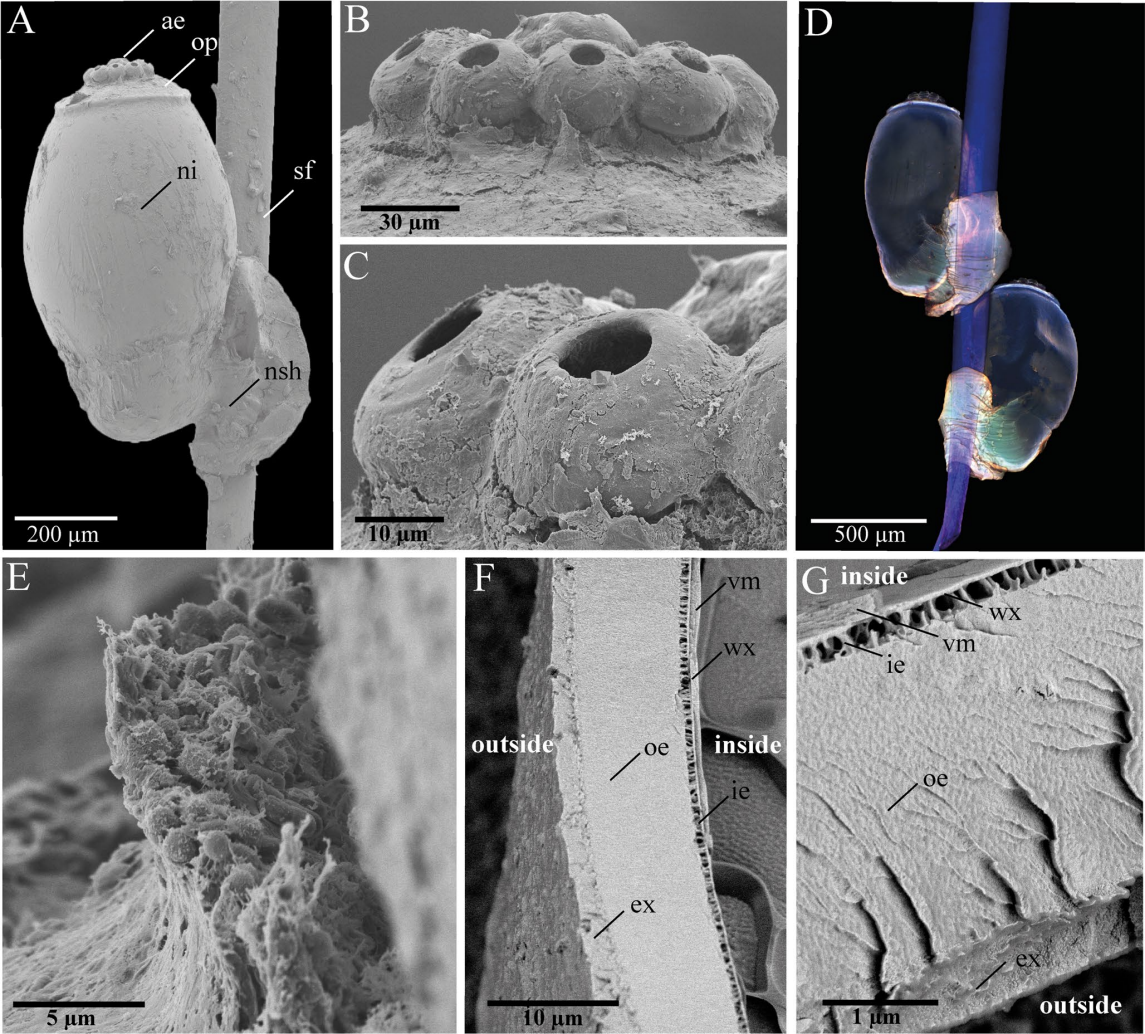
Morphology of seal louse nits. Scanning electron microscopy images of (**A**) a whole seal louse nit attached to seal fur, (**B**) and (**C**) aeropyles on top of the operculum, (**D**) confocal laser scanning microscopy image of two nits attached to seal fur, (**E**) the fibrous nit sheath at contact with the eggshell, and (**F**) and (**G**) cryofractures through the eggshell with visible layering. Abbreviations: ae (aeropyles); ex (exochorion); ie (inner endochorion); ni (nit); nsh (nit sheath); oe (outer endochorion); op (operculum); sf (seal fur); vm (vitelline membrane); wx (wax layer)

Seal hair is of a flattened shape (4.5 times as wide as thick) with a smooth surface and nits of *E. horridus* are firmly glued to the flat wide side of the hair (Fig. 2A, D). Thereby, the hair and the nit are surrounded by a sheath of solid material, which is called the nit sheath (Figs. 1A and 2A and E) (Carter 1990; Lapeere et al. 2005). This nit sheath consists of fibrous material, whose individual fibers usually do not merge smoothly into the surface of the hair or egg and show some fracture lines (Fig. 2E). The yellowish autofluorescence of this adhesive clearly sets it apart from the soft seal hair, which appears blue in the superimposed fluorescence maximum intensity projection images taken at different laser settings (Fig. 2D). The operculum stands out clearly from the rest of the nit and spherical formations with 40 μm diameter, the so-called aeropyles, are located at its uppermost tip. These aeropyles have large holes in their center (10 μm in diameter), which seem to extend to the operculum and are not covered by the nit sheath (Fig. 2B, C). On average, the nits each show 8–12 aeropyles. The nit itself appears bluish on the outside in the fluorescence projection, with the underlying layers shining through yellowish (Fig. 2D). The egg membrane consists of five visible layers, which are named from the inside to the outside as follows in the literature: vitelline membrane, dense wax layer, crystalline inner endochorion, amorphous outer endochorion, and fibrous exochorion (Fig. 2F, G) (Trougakos and Margaritis 2003). The vitelline membrane forms one of the thinnest layers with a thickness of approx. 0.3 μm, followed by the wax layer with a thickness of 0.1 μm. The inner endochorion is about 0.4 μm thick, while the outer endochorion forms the thickest layer at about 5 μm. The exochorion, which has a layer thickness of around 1 μm, is located on the very outside.

### Inner morphology of seal louse nits

**Fig. 3:**
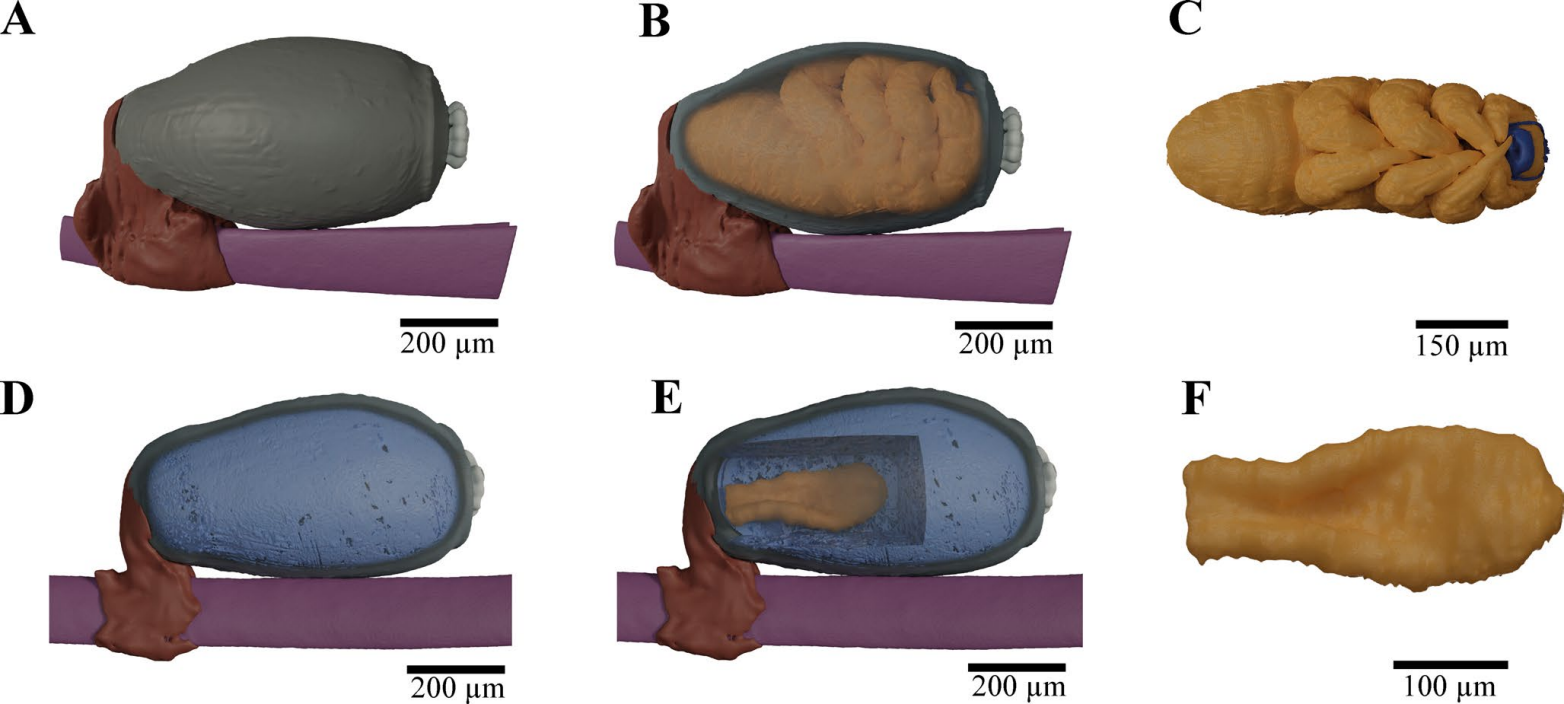
3D reconstruction of seal lice embryos inside their nits. **A**)-**C**) Seal louse nit including a louse embryo shortly before hatching. (**A**) Seal louse nit displayed as solid material with the nit shell in dark grey, the aeropyles in bright grey, the nit sheath in brown, and the seal hair in violet from lateral view. (**B**) Seal louse nit with transparent nit shell with visible louse embryo (gold) inside from lateral view. (**C**) Louse embryo without eggshell (gold) from ventral view including mask connected to the aeropyles (dark blue). **D**)**-F**) Seal louse nit including louse embryo at a very early stage of development. (**D**) Seal louse nit with transparent egg shell (dark grey) with visible yolk inside (light blue) from lateral view. (**E**) Seal louse nit with transparent eggshell (dark grey) and cut yolk (light blue) with visible embryo inside (gold) from lateral view. (**F**) Louse embryo (gold) without eggshell from ventral view

When looking at the inside of the nit, it can be seen that the louse embryo fills the entire inside of the nit shortly before hatching and has folded its legs tightly in front of its body (Fig. 3A-C). Its appearance is already strongly reminiscent of the first nymph stage and it is oriented with its head towards the operculum with its aeropyles. One structure, highlighted here in blue, is particularly striking: a mask-like structure that leads to the mouth opening of the louse on one side and with tubes on the other side through the operculum to the aeropyles (Fig. 3C). When looking at a nit with a louse in an earlier stage of development, one would notice that there is a large mass of yolk inside the egg in which the louse embryo floats (Fig. 3D-F). This embryo neither yet has any clearly recognizable differentiation of extremities, nor a mask-like structure, as can be seen in the later stage of development (Fig. 3F).

### Adhesive force of seal louse nits attached to seal fur

Adhesive force measurements of seal louse nits were performed in dry or wet condition on cut-off seal hairs, fixed with glue. Thereby, different orientations of the nits were measured: operculum oriented either upwards or downwards in dry or wet condition, resulting in four measurement scenarios. When accounting for the total maximum force needed for the removal of the nits from the seal hair in dry condition with the operculum oriented upwards (Du) averaged over 10 tested nits, it required 226.24 ± 77.68 mN, while for the downwards orientation of the operculum (Dd) it was 132.63 ± 121.84 mN. In wet condition, it took 148.00 ± 53.32 mN with the operculum oriented upwards (Wu) and 159.16 ± 62.37 mN for downwards orientation (Wd), respectively.

**Fig. 4:**
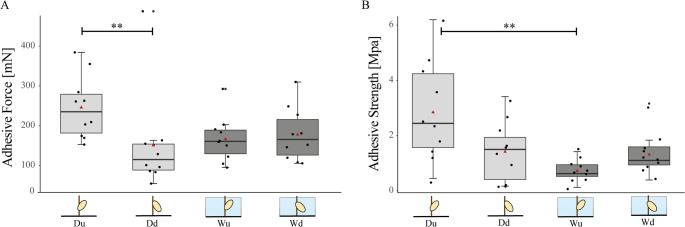
Adhesive force and adhesive strength of seal louse nits on seal hair. Boxplots showing the (**A**) pull-off force of seal louse nits on seal hair displayed as total force and (**B**) as adhesive strength (pull-off force divided by the contact area with the seal fur) of all measured nits in MPa. In total, 40 nits (10 for each orientation in wet or dry condition) were measured. The boxes indicate 25th and 75th percentiles, the line within the boxes represents the median, the red triangle shows the mean value, and whiskers (error bars) define the 10th and 90th percentiles

A Kruskal–Wallis test revealed a significant overall difference among the four treatments (H = 11.65, df = 3, *p* = 0.0087). Post hoc pairwise comparisons were performed using Dunn’s test with Bonferroni correction. A significant difference was detected between Du and Dd (p.adj = 0.0043), whereas all other comparisons orientations (Du-Wu; Du-Wd; Dd-Wu; Dd-Wd; Wu-Wd ) were not statistically significant (p.adj ≥ 0.05) (Fig. 4A).

In order to normalize the measured adhesive force to the contact area between the nit sheath and the hair surface, the corresponding adhesive strength was calculated. Adhesive strength averaged 2.86 ± 1.73 MPa for Du, 1.50 ± 1.01 MPa for Dd, 0.84 ± 0.35 MPa for Wu, and 1.41 ± 0.70 MPa for Wd. A Kruskal–Wallis test revealed a significant overall difference among the four treatments (H = 12.17, df = 3, *p* = 0.0068). Post hoc pairwise comparisons using Dunn’s test with Bonferroni correction showed a significant difference between Du and Wu (p.adj = 0.0030), whereas all other comparisons were not statistically significant (p.adj ≥ 0.05) (Fig. 4B).

## Discussion

This study provides new insights into the morphology, development, and attachment mechanisms of seal louse (*E. horridus*) nits, offering valuable information on the adaptations of these marine ectoparasites to their challenging underwater marine environment. Our findings reveal several key aspects of nit structure and function that may contribute to their survival on seal hosts.

### Nit morphology and respiratory adaptations

The detailed examination of nit morphology using advanced imaging techniques revealed several structural features that likely play important roles in embryo development and survival. The multi-layered eggshell consists of five distinct layers: vitelline membrane, wax layer (likely provides protection against water loss, water penetration, and bacterial attack due to its apparent hydrophobicity), inner endochorion (protects against temperature fluctuations, and enables the exchange of gases) and outer endochorion and exochorion (most likely for protection against mechanical stress due to their density) (Fig. 2F, G). Conversely, the eggshell facilitates the exchange of gases and helps in regulating appropriate moisture levels (Margaritis et al. 1980; Chapman 1998; Al-Dosary et al. 2010). The presence of aeropyles on the operculum is particularly noteworthy, as these structures appear to form a direct connection to the developing embryo inside the nit (Figs. 2B and C and 3). Consequently, these formations are composed of a network of openings that narrow from a wider entrance to a smaller exit. This suggests that the aeropyles, which consist of complex, labyrinthine channels, may prevent the passage of water but allow gas exchange (Berman and Firstenberg 1979; Berman et al. 1980). Furthermore, the diameters of the aeropyles are extremely small, which makes it even more difficult for water to enter. Even with a short immersion, liquids can only penetrate the egg shell with difficulty due to the surface tension and the apparently hydrophobic narrow channels (Akhoundi et al. 2024). Consequently, air and oxygen can pass through, while liquids are blocked by the capillary effect (Berman and Firstenberg 1979; Berman et al. 1980). This in turn may reduce the risk of water penetration during host dives. With an average of 8–12 aeropyles per nit, seal lice have a similar number of aeropyles as human head lice (5–12 aeropyles), but fewer than body lice (10–16) and pubic lice (13–18). However, the aeropyles of seal lice (40 μm) are on average only about as large as those of pubic lice (41.2 μm), whereas those of human head lice (51.1 μm) and body lice (49.1 μm) are significantly larger (Akhoundi et al. 2024). In combination with the resulting smaller openings of the aeropyles, this could also be an adaptation to the underwater life of the seal lice, in order to minimize the penetration of water. The rather small number of aeropyles in combination with their comparatively small size could also be an indication that the embryos of the seal lice in the nits generally have a lower oxygen requirement than their terrestrial relatives or, similar to their adult specimens, can shut down their metabolism accordingly, if necessary, or can breathe through the egg chorion or via cutaneous gas exchange (Leonardi and Lazzari 2014; Leonardi et al. 2020).

The mask-like structure observed in late-stage embryos, connecting the mouth opening to tubes leading through the operculum to the aeropyles, further supports the hypothesis of a specialized respiratory mechanism, that has never been, to our knowledge, described for any other insect before (Fig. 3C). However, this interpretation is currently based solely on morphological evidence obtained by micro-CT and SEM. Direct measurements of oxygen flux or permeability were not performed, and the proposed respiratory function therefore remains hypothetical and requires physiological validation in future studies.

We observed that the mask-like structure was absent in early-stage embryos, indicating its development may be crucial for the transition to a more advanced developmental stage capable of surviving in an aquatic environment (Burkhart and Burkhart 2005) (Fig. 3F). In the earlier stage embryo, the lice might breathe by diffusive oxygen uptake through the eggshell or the aeropyles, as their tracheal system is not yet sufficiently developed (Wigglesworth and Beament 1950; Woods and Hill 2004). The egg yolk contains essential nutrients, including proteins, lipids, and carbohydrates, which serve as a source of nourishment and facilitate the embryo’s development. Additionally, these components presumably act as a medium for transporting oxygen to the growing embryo (Nagy and Grbić 2009). As soon as the embryo grows larger and the tracheal system gradually develops, this system becomes necessary for efficient oxygen distribution. Only from this stage the embryo can actively distribute larger quantities of oxygen (Wigglesworth 1953; Kaars 1981). However, even with a developed tracheal system, the lice cannot remain underwater indefinitely long without an external oxygen supply. Previous studies have already shown that the embryos within the eggs do not survive exposure to seawater for long time, but the N1 nymphs in their early developmental stage are also less likely to survive, if they are not regularly exposed to atmospheric oxygen possibly due to an underdeveloped tracheal system at earlier developmental stages (Murray and Nicholls 1965; Murray et al. 1965; Aznar et al. 2009; Leonardi and Lazzari 2014; Polilov 2015, 2016). This finding let us to support the hypothesis that the tracheal volume and its capacity for air storage play a role in the underwater breathing of the seal louse embryo (Preuss et al., 2025a). It can therefore be assumed that the lice inside the nits can simply withstand a short period of time under water without drowning. This vulnerability could explain, why some studies have reported lower viability of eggs found on seals during non-breeding periods, when the animals spend more time in water (Murray et al. 1965; Leonardi and Lazzari 2014).

### Nit attachment and water resistance

Our force measurements provide quantitative data on the adhesive force of seal louse nits to seal hair under different conditions. We could only find significant differences between adhesive forces of nits in dry condition with different operculum orientations (Fig. 4).

To assess the forces impacting lice during seal dives, we computed the drag force experienced by a single louse nit on the most exposed seal area using the following equation (Büscher et al. 2022):

$$\:D=\:\frac{{C}_{d}*\rho\:*S*{v}^{2}}{2}$$

The drag coefficient (*C*_*d*_) of a sphere is derived from the calculated Reynold’s number for a seal louse nit in water. The fluid density ($\:\rho\:$) of water is 1000 kg*m^-3^, *S* represents the area opposing water flow, and *v* denotes the seal’s swimming velocity of 4.9 m*s^-1^ (Williams and Kooyman 1985) (Supplementary Data S3). The resulting drag force of 0.00566 mN, when compared to the significantly higher adhesive force that the seal louse nit can generate, indicates that the adhesive force of nits is in dry condition approximately 39,972 times (operculum oriented upwards; 226.24 mN) or 23,433 times, respectively, (operculum oriented downwards; 132.63 mN) greater than the drag force it encounters. However, this calculation would assume that the nit is transferred directly from the dry state to the wet state, which would only be the case when the seal dives into the water after being a while exposed to air. When considering the drag force under water in comparison to the adhesive force of the nits under water, this reveals that the adhesive force is 26,148 times (operculum oriented upwards; 148.00 mN) and 28,120 times (operculum oriented downwards; 159.16 mN) greater than the drag force acting on the nit.

Despite being a highly simplified estimate, we can conclude that the seal louse nit is adequately strong glued to the seal hair even during rapid, deep dives as it is also additionally protected by the seal fur itself (drag forces are much lower in the boundary layer of water situated between hairs). When comparing these values to values measured for human head louse nits (*Pediculus humanus capitis*), it is noticeable that the maximum values of the forces for the nits of the seal louse are about twice as high as those for the human head louse. This could be interpreted as a special adaptation to the marine environment or also as a result of the different measurement methodology (Lapeere et al. 2005). The different values based on the orientation of the nits on the hair could also be understood as an adaptation to the lifestyle and environment of the lice: the lice usually lay their eggs with the operculum oriented towards the tip of the seal’s hair, so that the operculum points away from the seal’s skin (Figs. 1 and 2). This is probably due to the fact that they want to attach the eggs as basally as possible to the hair root, in order to offer their eggs the best protection from the seal’s fur (Preuss et al. 2024), and additionally, the louse first spreads the adhesive on the hair and then positions the egg on the adhesive in such a way that the operculum is not covered by it (Carter 1990; Kim et al. 2021).

However, orientation only appears to have a significant influence on adhesive force in a dry environment. In a humid environment, we were unable to detect any significant difference in this respect (Fig. 4). This could possibly be related to the fact that the seal hairs swell on contact with water and thus become thicker overall, which could prevent the nits from slipping off (Lapeere et al. 2005). In human hair, the keratinized cells of the hair cuticle spread out between 50 and 150% when hydrated (O’Connor et al. 1995), but in seal hair, this process has not been analyzed yet, but it is reasonable to assume that the situation is similar there. When dry and with upwards oriented operculum, we observed that the nits did not normally separate from the adhesive when we pulled them off the hair, but that the adhesive no longer adhered to the seal hair at the maximum adhesive force and we thus pulled the nits and adhesive off the hair (Supplementary Material S4A). In contrast, when the operculum was oriented downwards, the nit was torn away from the adhesive and we could find adhesive residues on both the hair and the nit. Although seal hairs have a fairly smooth surface (Preuss et al. 2024), they become narrower towards the tip and since we pulled them off with the operculum pointing downwards towards the broad base of the seal hair, the adhesive could not simply slide off the hair but was torn. When wet, the seal hair presumably swells under water to a certain degree, so that we were able to observe a similar phenomenon as in the dry state with the operculum oriented downwards: the adhesive was torn in the middle and we were able to find residuals on both the nit and the hair (Supplementary Material S4B & C). This indicates that the adhesive bond to the seal hair remains strong even under wet conditions, even if it seems to be even stronger when dry with the operculum oriented upwards, but this could also be related to the method of removing the nit from the seal hair.

**Fig. 5:**
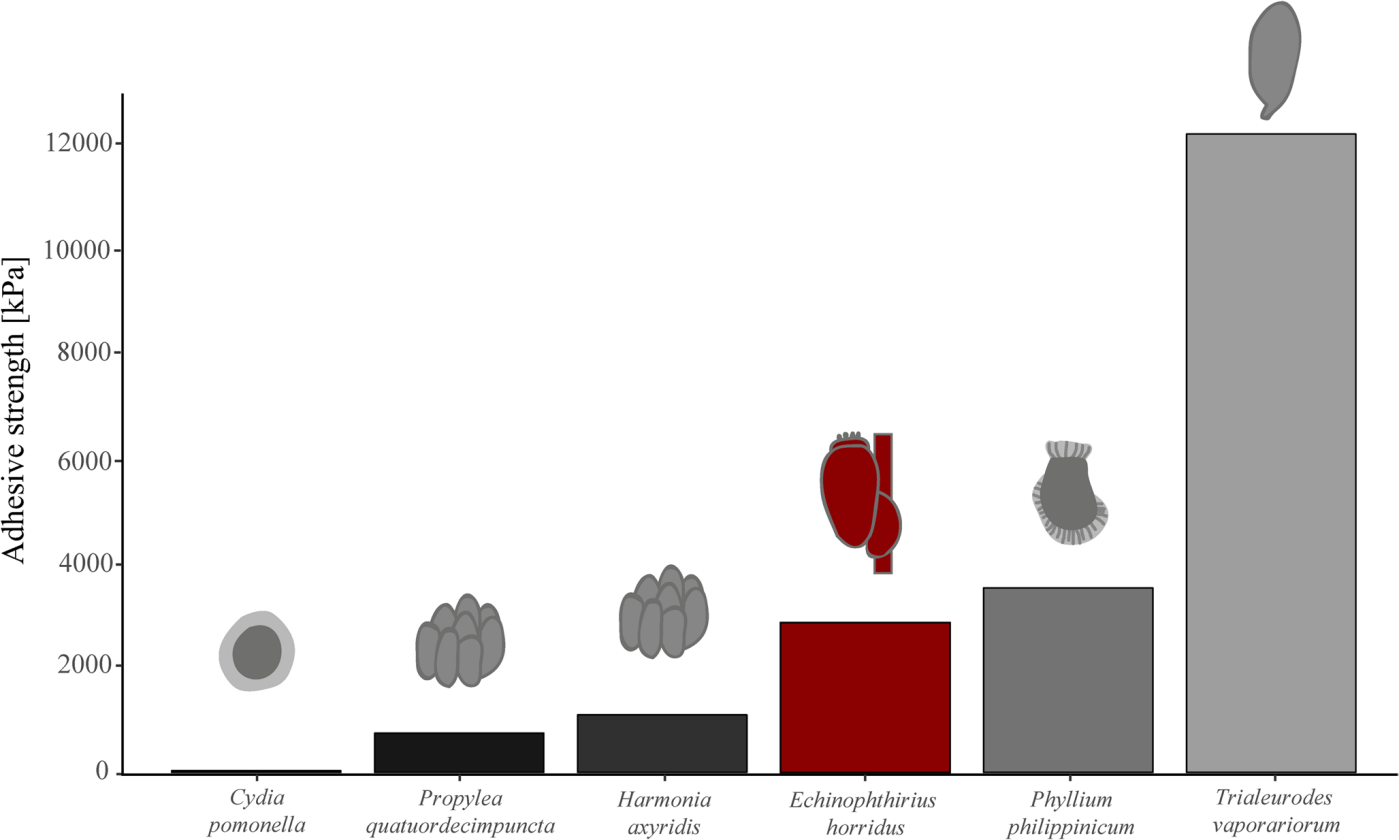
Comparison of adhesive strengths of eggs on various surfaces in different insect species. X-axis displays insect species sorted by their egg adhesive strength in ascending order. Y-axis displays the adhesive strength in kPa. A detailed list of all reported adhesive strengths including associated references can be found in the Supplementary Material S5

When comparing the adhesive strength of seal louse nits on seal hair with adhesive strength of other insect eggs on various surfaces, it becomes clear that the nits have a fairly strong adhesion to their substrate (Fig. 5). For example, when looking at the eggs of the codling moth, *Cydia pomonella*, it is noticeable that it only has a relatively low adhesive strength of about 31 kPa on a 3 μm rough substrate, which is sufficient for adhesion to the leaves of apple trees even in rain and wind (Al Bitar et al. 2012). The eggs of the two ladybird species, *Propylea quatuordecimpunctata* and *Harmonia axyridis*, have adhesive strengths of around 750 and 1100 kPa on smooth and 1 μm rough polishing paper respectively (Salerno et al. 2022). All these three insect species, as well as the seal louse, *E. horridus*, with an adhesive strength of 2860 kPa in the dry state with upward operculum, rely on protein-based glue as the only adhesion mechanism. In lice, the adhesive material forming the nit sheath has recently been linked to specific louse nit sheath proteins (LNSPs), first identified using the genome of the human body louse and subsequently compared across several primate lice species (Burkhart 1999; Park et al. 2019, 2025; Kim et al. 2021). Whether homologous adhesive proteins occur in *E. horridus* remains unknown, as genomic resources for this species are still limited. If similar proteins are present in echinophthiriid lice, it would be interesting to investigate whether modifications in their amino acid composition or cross-linking properties contributed to the increased water tolerance and mechanical stability required for egg attachment in a marine environment.

Leaf insect eggs, on the other hand, have a higher adhesive strength of about 3527 kPa, but they do not use purely adhesive-based attachment: The eggs of *Phyllium philippinicum* use a water-activated adhesive and unfolding pinnae (hair-like structures) to attach securely to various surfaces. This mechanism is highly efficient because it ensures egg stability in unpredictable environments and allows reversible attachment for optimal positioning and survival (Büscher et al. 2020a, b, 2023; Büscher and Gorb 2022). Another very efficient mechanism can be found in the greenhouse whitefly: the greenhouse whitefly (*Trialeurodes vaporariorum*) secures its eggs to plant leaves by inserting a stalk-like structure, the pedicel, into the leaf’s epidermis. This pedicel, enveloped in a proteinaceous secretion from the colleterial glands, functions like a wall plug in sealing cement, forming a composite bond through mechanical interlocking, friction, and adhesion (Voigt et al. 2019). So, when looking at the purely adhesive-based mechanisms, the seal louse has some of the highest adhesive strength values ever measured, which are sufficient to ensure secure attachment of nits under a wide range of outdoor conditions (Figs. 4 and 5). However, if other adhesion mechanisms with additional hairs or insertions in the epidermis of leaves are used, these are even more efficient, but are particularly suitable for adhesion to substrates of varying roughness (Voigt et al. 2019; Büscher et al. 2020a, b, 2023; Büscher and Gorb 2022), which is not necessary for the seal louse, as the hairs of the harbor seal are very smooth overall (Preuss et al. 2024). Although the number of measurements per treatment was limited (*n* = 10), the observed patterns were consistent across samples. Future studies with larger sample sizes could further refine quantitative comparisons of adhesive performance.

It should be considered that the specimens analyzed in this study were stored frozen prior to examination in order to prevent tissue degradation, and specimens used for synchrotron micro-CT were additionally stored in 70% ethanol before scanning. Freezing and ethanol storage may influence protein conformation, fine-scale structural features, and the mechanical properties of biological materials. Thus, both morphological observations and absolute adhesion values should be interpreted within the context of the present experimental conditions. However, because all samples within each analytical approach were subjected to identical preservation protocols, comparative analyses and relative differences between experimental groups remain robust. Future studies using freshly collected material or in vivo measurements would further refine the understanding of structural and adhesive performance under natural conditions.

### Evolutionary implications

The adaptations observed in *E. horridus* nits, including their potential specialized respiratory structures and attachment mechanism, provide insights into the evolutionary pressures faced by these ectoparasites, as their hosts made transition from land to the sea during the Miocene (Anderson 1982; Raga et al. 2009; Rybczynski et al. 2009). The development of a respiratory system that may be capable of functioning in both air and water and an attachment mechanism that maintains its effectiveness in dry and wet conditions demonstrates the remarkable adaptability of these insects to an extremely challenging niche.

These findings also highlight the complex interplay between parasite and host life cycles. The potential ability of seal louse nits to survive underwater periods while remaining attached to their host’s fur may facilitate reproduction throughout the year (Herzog et al. 2024a, b). However, the presence of nits throughout the year does not necessarily indicate successful development under prolonged immersion. Ecological factors such as water temperature and host diving behavior may strongly influence embryo survival and reproductive success. In particular, prolonged diving periods may reduce the likelihood of nits developing to hatching, potentially explaining the lower parasite loads observed during non-breeding periods when seals spend more time in water (Murray et al. 1965; Aznar et al. 2009; Leonardi and Lazzari 2014).

For the future, comparative studies with other echinophthiriid species parasitizing different marine mammal hosts could reveal the diversity of adaptations within this family and provide further insights into the evolution of marine ectoparasitism.

## Conclusion

This study investigated the morphology, development, and attachment of seal louse nits in order to better understand how these ectoparasites cope with the challenges of a marine environment. Morphological analyses revealed the multilayered structure of the nit eggshell as well as the presence of aeropyles and associated microstructures that may facilitate gas exchange while limiting water intrusion. Developmental observations showed that later embryonic stages possess a distinct mask-like structure associated with the aeropyles. In addition, attachment experiments demonstrated that the nit sheath forms a strong and water-tolerant bond with seal hairs, indicating that detachment under wet conditions is unlikely to represent a major constraint for reproduction.

Taken together, these findings demonstrate that seal louse nits possess a range of morphological and functional adaptations that enable their persistence in a marine environment. The identification of potential respiratory structures and water-tolerant attachment mechanisms provides a foundation for understanding how these ectoparasites have successfully adapted to marine mammals during their transition from terrestrial to aquatic lifestyles. Our findings not only contribute to our knowledge of parasite–host interactions in marine ecosystems but also offer potential inspiration for the development of novel biomimetic adhesives based on nature’s solutions.

## Supplementary Information

Below is the link to the electronic supplementary material.

Supplementary Material 1 (XLSX 31.5 KB)

Supplementary Material 2 (DOCX 17.4 KB)

Supplementary Material 3 (DOCX 18.1 KB)

Supplementary figure 1 (5.03 MB)

High Resolution Image (TIF 50.4 MB)

Supplementary Material 5 (XLSX 12.8 KB)

Supplementary Material 6 (PDF 125 KB)

## Data Availability

The data supporting the findings of this study are available in the Supplementary Material. CT data can be accessed via 10.6084/m9.figshare.29712767.
