# Supplementary material for: Adaptations of seal louse nits to underwater life: morphology, respiration and attachment
Source: Naturwissenschaften. 2026 Apr 10;113(3):50. doi: 10.1007/s00114-026-02095-2 (PMC13068745; doi:10.1007/s00114-026-02095-2)
Supplement: Supplementary file 2 — Supplementary Material 2 (DOCX 17.4 KB) [file 114_2026_2095_MOESM2_ESM.docx]

**Supplementary Material S2**

########################################################################################################

This R-script is part of the manuscript "Attachment of seal louse nits"

+++++++++ Kruskal-Wallis-Test Fad +++++++++++++

########################################################################################################

# Clear workspace

rm(list = ls())

library(ggplot2)

library(tidyverse)

library(ggpubr)

library(rstatix)

# Import dataset

library(readxl)

S1_Nit_Attachment_Force <- read_excel("S1_Nit_Attachment_Force.xlsx",

sheet = "Summary_dataset")

View(S1_Nit_Attachment_Force)

attach(S1_Nit_Attachment_Force)

# Data preparation

S1_Nit_Attachment_Force <- S1_Nit_Attachment_Force %>%

gather(key = "Iteration", value = "Fad_in_mN", D1, D2, W1, W2) %>%

convert_as_factor(Iteration)

data.frame(head(S1_Nit_Attachment_Force, 40))

# Summary statistics

S1_Nit_Attachment_Force %>%

group_by(Iteration) %>%

get_summary_stats(Fad_in_mN, type = "mean_sd")

# Check for outliers

S1_Nit_Attachment_Force %>%

group_by(Iteration) %>%

identify_outliers(Fad_in_mN)

# Normality assumption

S1_Nit_Attachment_Force %>%

group_by(Iteration) %>%

shapiro_test(Fad_in_mN) # not normally distributed -> Kruskal Wallis Test

# Kruskal Wallis Test

kruskal.test(S1_Nit_Attachment_Force$Fad_in_mN~S1_Nit_Attachment_Force$Iteration)

# Post-hoc Test: Dunn's

dunn_test(Fad_in_mN~Iteration, data=S1_Nit_Attachment_Force,

p.adjust.method = "bonferroni")

############################################################################################################################################

This R-script is part of the manuscript "Attachment of seal louse nits"

+++++++++ Boxplot Force in mN +++++++++++++

###########################################################################################################################################

# Clear workspace

rm(list = ls())

library(ggplot2)

# Import dataset

library(readxl)

S1_Nit_Attachment_Force <- read_excel("F:/Manuskripte/Nits/S1_Nit_Attachment_Force.xlsx",

sheet = "Summary_datset")

View(S1_Nit_Attachment_Force)

attach(S1_Nit_Attachment_Force)

# Make boxplot

pd = position_dodge(width = 1.1)

jitter <- position_jitter(width = 0.15, height = 0.15)

p <- ggplot(S1_Nit_Attachment_Force, aes(x=Iteration, y=Fad_in_mN)) +

stat_boxplot(geom='errorbar', position = pd, width=0.1) +

geom_boxplot(width = 0.3, position=position_dodge(width = 1.1)) +

geom_point(position = jitter) +

stat_summary(fun = mean, geom = "point", color = "firebrick", shape = 17, size = 2, position = position_dodge(width = 1.1)) +

scale_fill_manual(values=c("#999999", "#E69F00")) +

theme_classic() +

labs(x = "Iteration", y = "Force in mN") +

theme(plot.title = element_text(hjust = 0.5, size = 16, face = "bold"), plot.caption = element_text(hjust = 0, size = 10), plot.tag = element_text(size = 16, face = "bold")) # adjust title and caption position

p

############################################################################################################################################

This R-script is part of the manuscript "Attachment of seal louse nits"

+++++++++ Boxplot Force σ +++++++++++++

###########################################################################################################################################

# Clear workspace

rm(list = ls())

library(ggplot2)

# Import dataset

library(readxl)

Nit_attachment <- read_excel("F:/Manuskripte/Nits/S1_Nit_Attachment_Force.xlsx",

sheet = "Summary_datset2")

View(S1_Nit_Attachment_Force)

attach(S1_Nit_Attachment_Force)

# Make boxplot

pd = position_dodge(width = 1.1)

jitter <- position_jitter(width = 0.15, height = 0.15)

p <- ggplot(S1_Nit_Attachment_Force, aes(x=Iteration, y=σ_c)) +

stat_boxplot(geom='errorbar', position = pd, width=0.1) +

geom_boxplot(width = 0.3, position=position_dodge(width = 1.1)) +

geom_point(position = jitter) +

stat_summary(fun = mean, geom = "point", color = "firebrick", shape = 17, size = 2, position = position_dodge(width = 1.1)) +

scale_fill_manual(values=c("#999999", "#E69F00")) +

theme_classic() +

labs(x = "Iteration", y = "σ") +

theme(plot.title = element_text(hjust = 0.5, size = 16, face = "bold"), plot.caption = element_text(hjust = 0, size = 10), plot.tag = element_text(size = 16, face = "bold")) # adjust title and caption position

p

########################################################################################################

This R-script is part of the manuscript "Attachment of seal louse nits"

+++++++++ Barplot comparison insect egg attachment +++++++++++++

########################################################################################################

# Clear workspace

rm(list = ls())

library(ggplot2)

# Import dataset

library(readxl)

S5_Comparison_of_egg_attachment_force <- read_excel("F:/Manuskripte/Nits/Manuscript/fertig/S5_Comparison_of_egg_attachment_force.xlsx",

sheet = "Tabelle2")

View(S5_Comparison_of_egg_attachment_force)

attach(S5_Comparison_of_egg_attachment_force)

# Make barplot

egg.data <- data.frame(

egg_ID <- c(1:6),

Species_Name <- c("Cydia_pomonella","Propylea_quatuordecimpuncta","Harmonia_axyridis","Echinophthirius_horridus","Phyllium_philippinicum", "Trialeurodes_vaporariorum"),

Adhesive_strength <- c(31, 750, 1100, 2860, 3527, 12200))

# Plot the bar chart

barplot(Adhesive_strength,names.arg=Species_Name,xlab="Species",

ylab="Adhesive strength",col="black")
