# Supplementary material for: Adaptations of seal louse nits to underwater life: morphology, respiration and attachment
Source: Naturwissenschaften. 2026 Apr 10;113(3):50. doi: 10.1007/s00114-026-02095-2 (PMC13068745; doi:10.1007/s00114-026-02095-2)
Supplement: Supplementary file 3 — Supplementary Material 3 (DOCX 18.1 KB) [file 114_2026_2095_MOESM3_ESM.docx]

**Supplementary Material S3**

**S 3)** Table of parameters for the estimation of the drag force a single seal louse nit, *E. horridus*, is exposed on the surface of a swimming seal.

| **Symbol** | **Parameter** | **Value** | **Unit** |
| --- | --- | --- | --- |
| *v* | Swimming speed (seal) | 4.9 | m/s |
| *S* | Flow resisting area (*E. horridus*) | 1,96×10^−7^ | m^2^ |
| *C_d_* | Drag coefficient (sphere) | 0.0024 |  |
| *p* | Fluid density (water) | 1000 | kg/m^3^ |
| *D* | Drag force | 0.00566 | mN |
| *F* | Attachment force nit (Du) | 226.24 | mN |
| *F* | Attachment force nit (Dd) | 132.63 | mN |
| *F* | Attachment force nit (Wu) | 148.00 | mN |
| *F* | Attachment force nit (Wd) | 159.16 | mN |
|  | Attachment force/drag force (Du) | 39971.73 |  |
|  | Attachment force/drag force (Dd) | 23432.86 |  |
|  | Attachment force/drag force (Wu) | 26148.41 |  |
|  | Attachment force/drag force (Wd) | 28120.14 |  |

The attachment forces of *E. horridus* nits on seal fur are 39972 (Du), 23433 (Dd), 26148 (Wu), and 28120 times stronger than the drag force generated at the most exposed area of the seal at a swimming speed of 4.9 m/s.
