# Supplementary figures and images for: Adaptations of seal louse nits to underwater life: morphology, respiration and attachment
Source: Naturwissenschaften. 2026 Apr 10;113(3):50. doi: 10.1007/s00114-026-02095-2 (PMC13068745; doi:10.1007/s00114-026-02095-2)

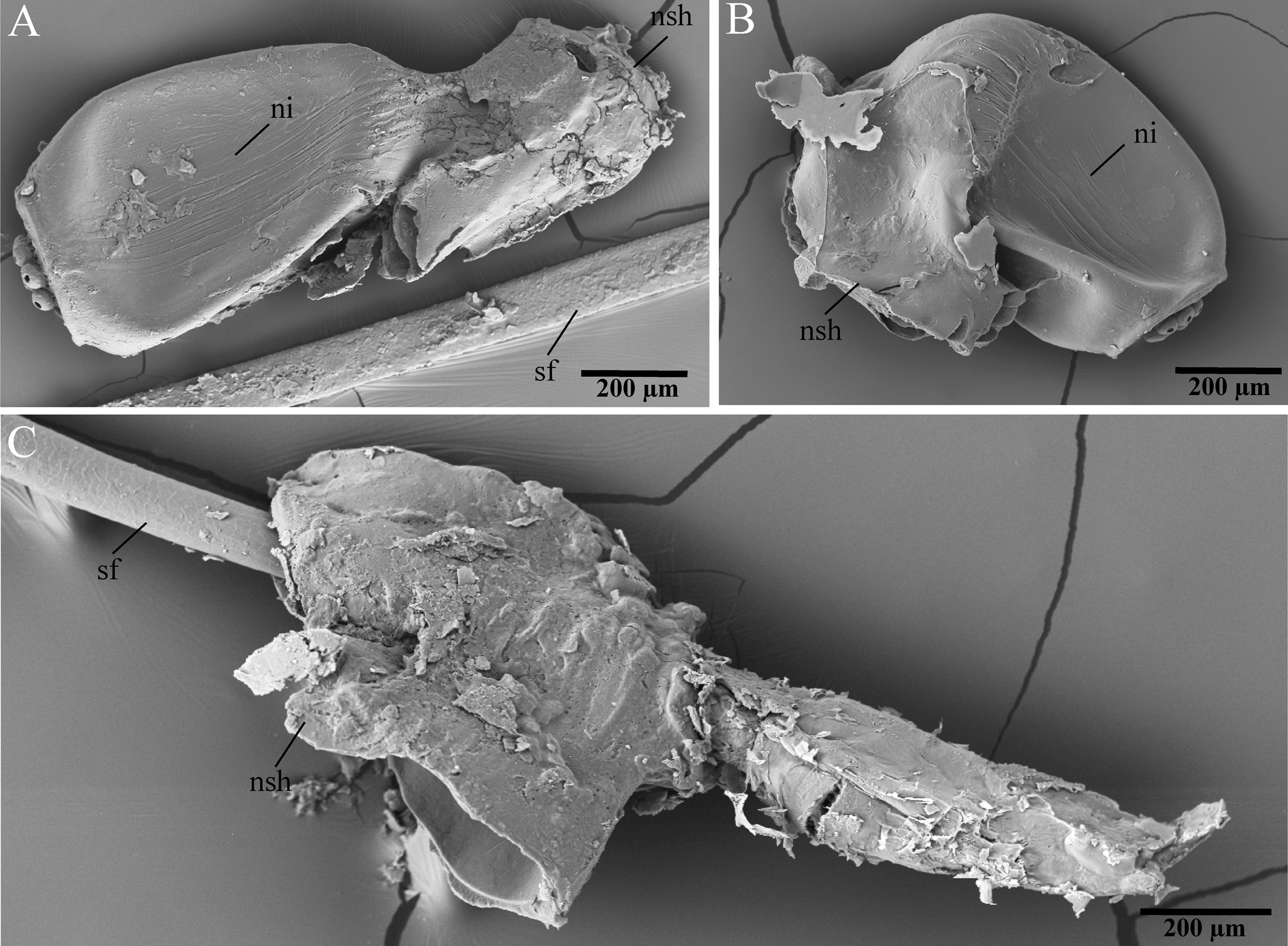

Supplement: Supplementary file 4 — (5.03 MB) [file 114_2026_2095_Fig6_ESM.png]

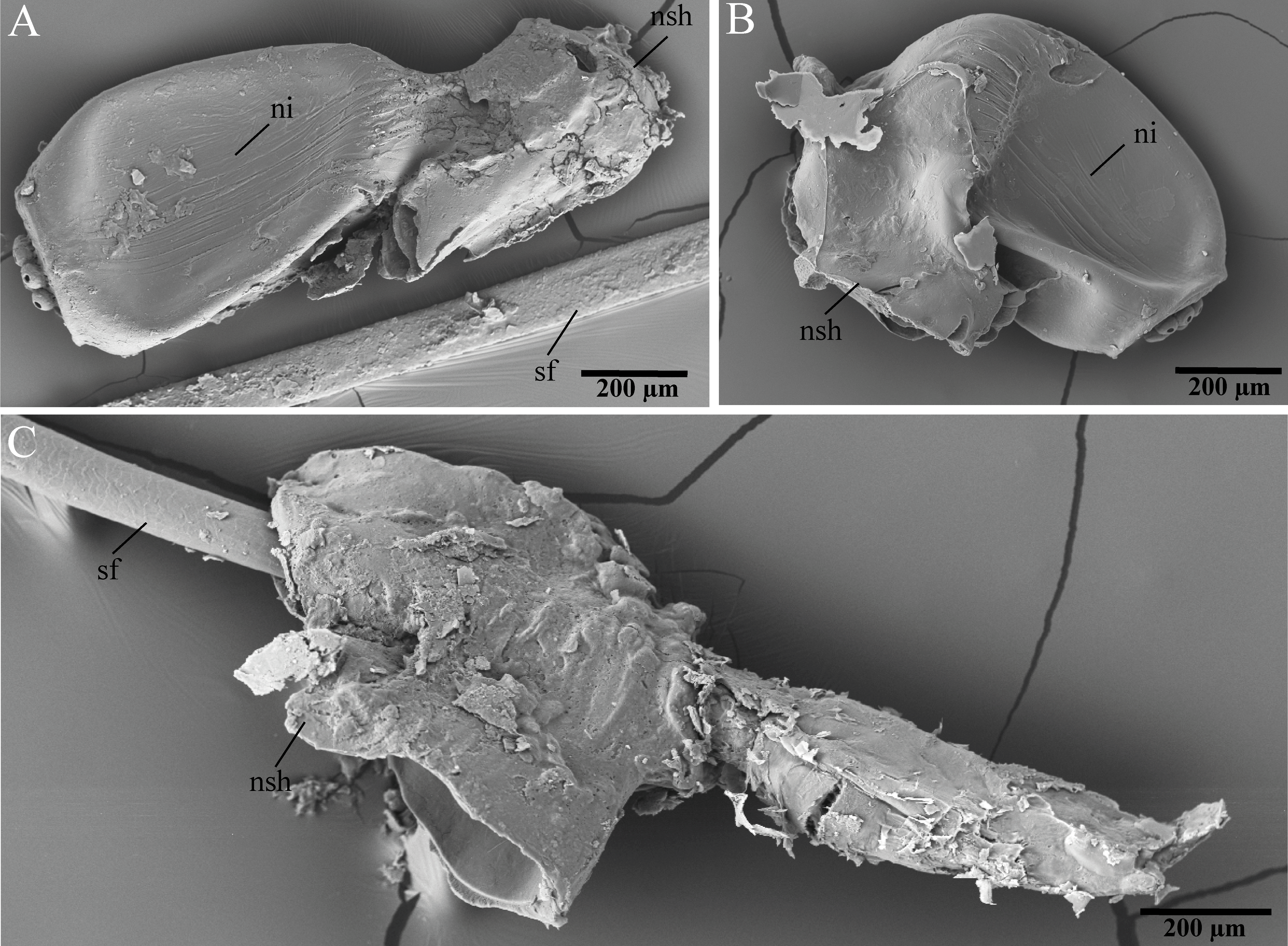

Supplement: Supplementary file 5 — High Resolution Image (TIF 50.4 MB) [file 114_2026_2095_MOESM4_ESM.tif]
