# Supplementary material for: Adaptations of seal louse nits to underwater life: morphology, respiration and attachment
Source: Naturwissenschaften. 2026 Apr 10;113(3):50. doi: 10.1007/s00114-026-02095-2 (PMC13068745; doi:10.1007/s00114-026-02095-2)
Supplement: Supplementary file 7 — Supplementary Material 6 (PDF 125 KB) [file 114_2026_2095_MOESM6_ESM.pdf]

Supplementary Materials

S1) Dataset for measured nit attachment forces in both directions and in wet and dry condition.

S2) R-scrips used for graphs and statistics.

S 3) Table of parameters for the estimation of the drag force a single seal louse nit, *E. horridus*, is exposed on the surface of a swimming seal.

Symbol	Parameter	Value	Unit
v	Swimming speed (seal)	4.9	m/s
S	Flow resisting area (*E. horridus*)	$1{,}96\times10^{-7}$	m^2
C_d	Drag coefficient (sphere)	0.0024	
p	Fluid density (water)	1000	kg/m^3
D	Drag force	0.00566	mN
F	Attachment force nit (Du)	226.24	mN
F	Attachment force nit (Dd)	132.63	mN
F	Attachment force nit (Wu)	148.00	mN
F	Attachment force nit (Wd)	159.16	mN
	Attachment force/drag force (Du)	39971.73	
	Attachment force/drag force (Dd)	23432.86	
	Attachment force/drag force (Wu)	26148.41	
	Attachment force/drag force (Wd)	28120.14	

The attachment forces of *E. horridus* nits on seal fur are 39972 (Du), 23433 (Dd), 26148 (Wu), and 28120 times stronger than the drag force generated at the most exposed area of the seal at a swimming speed of 4.9 m/s.

S4) Scanning Electron Microscopy images of A) nit and nit sheath completely pulled off the seal hair under dry condition with operculum oriented upwards, B) nit and parts of the nit sheath pulled off the seal hair when the operculum was oriented downwards under dry condition and in both orientations under wet condition, and C) seal hair with residuals of nit sheath on its surface when the operculum was oriented downwards under dry condition and in both orientations under wet condition. Abbreviations: ni (nit), nsh (nit sheath), sf (seal fur).

S5) Dataset for measured attachment forces for various insect eggs on different surfaces based on literature values.
